# The Association of Surrogates of Insulin Resistance with Hyperuricemia among Middle-Aged and Older Individuals: A Population-Based Nationwide Cohort Study

**DOI:** 10.3390/nu15143139

**Published:** 2023-07-14

**Authors:** Yutong Han, Zonglei Zhou, Yuge Zhang, Genming Zhao, Biao Xu

**Affiliations:** 1 Department of Epidemiology, School of Public Health, Fudan University, Shanghai 200032, China; ythan22@m.fudan.edu.cn (Y.H.); zonglei_zhou@fudan.edu.cn (Z.Z.); yuge_z2015@163.com (Y.Z.); gmzhao@shmu.edu.cn (G.Z.); 2 Key Laboratory of Health Technology Assessment, National Health Commission of the People’s Republic of China (Fudan University), Shanghai 200032, China

**Keywords:** insulin resistance, hyperuricemia, cohort study

## Abstract

The triglyceride–glucose (TyG) index, triglyceride-to-high-density-lipoprotein-cholesterol (TG/HDL-C) ratio, metabolic score for insulin resistance (METS-IR) and TyG with body mass index (TyG-BMI) have been proposed as indicators of insulin resistance (IR). This study aimed to explore the association between these IR surrogates and their longitudinal variation with the development of hyperuricemia in a middle-aged and older Chinese population. Data from the China Health and Retirement Longitudinal Study (CHARLS) was used to identify 5269 participants aged ≥45 years. Logistic regression was used to assess the effect of IR surrogates and their variations on the risk of hyperuricemia. After four years of follow-up, 517 (9.81%) participants developed incident hyperuricemia. Increased baseline values of TyG, TG/HDL, METS-IR, and TyG-BMI were all significantly associated with higher risks of hyperuricemia. Compared to individuals with maintained low levels of IR surrogates, those with low-to-high and maintained high variation patterns had a significantly higher risk of hyperuricemia. These four IR surrogates have comparable predictive ability for hyperuricemia. This study provides evidence of the associations between IR and hyperuricemia. Early intervention among middle-aged and elderly Chinese individuals with high IR levels may effectively reduce the burden of hyperuricemia.

## 1. Introduction

Hyperuricemia is characterized by an elevation of uric acid (UA) levels in the peripheral blood, which can be attributed to either excessive production or insufficient renal excretion of UA. Despite its adverse impacts on gout and chronic kidney disease [1], growing evidence suggests that UA levels are positively correlated with various other metabolic diseases, including cardiovascular disease, type 2 diabetes mellitus, hypertension, and non-alcoholic fatty liver disease [2,3,4]. The prevalence of hyperuricemia varies across geographical areas. For example, it is 20.1% in the United States [5] and 11.4% in Korea [6]. A national representative survey conducted in China from 2015 to 2017 found that the total prevalence of hyperuricemia was 15.1% [7]. Additionally, the prevalence and risk of hyperuricemia increase significantly with age [8]. With the current aging population, the burden of this disease will be rising. Due to its increasing global prevalence and clinical significance, hyperuricemia has become a noteworthy public health issue [9,10]. Hence, early identification and management of hyperuricemia may be a promising way to reduce the disease burden and prevent adverse outcomes associated with this condition.

Insulin resistance (IR) is a condition defined as an impaired physiologic response of target tissues, such as liver, muscle, and adipose tissue, to insulin stimulation [11]. IR plays an important role in the pathogenesis of many metabolic diseases. Epidemiological and mechanistic research has illustrated the correlation between IR and serum urate concentration [12,13,14]. In line with these findings, research has shown that reducing IR can lower serum UA values and decrease the risk of gout [15], indicating that the level of IR may serve as a reliable predictor of hyperuricemia development. The gold standard for assessing IR is the hyperinsulinemic–euglycemic clamp method. This approach, however, has limited usage in clinical practice due to its complexity and high cost [11]. By contrast, several IR indicators that are not based on insulin, known as IR surrogates, which can be determined using regular biochemical test indexes, have been proposed to reflect IR levels, including triglyceride–glucose (TyG) index [16], triglyceride-to-high-density -lipoprotein-cholesterol (TG/HDL-C) ratio [17], TyG with body mass index (TyG-BMI) [18] and metabolic score for IR (METS-IR) [19].

Previous epidemiological studies have demonstrated the significant association between individual indicators of IR, such as TyG index or TG/HDL-C, and hyperuricemia [20,21]. However, most of these studies have used cross-sectional study designs. Longitudinal studies that use data from nationally representative research, which could further corroborate these discoveries, are limited, especially among the Chinese population. Additionally, it is not fully understood whether dynamic changes in these indices over time could impact the development of hyperuricemia. Furthermore, it is disputed which IR surrogates could most accurately forecast the incident of hyperuricemia [22,23]. To address these knowledge gaps, we investigated the associations between four surrogate markers of IR and their changes on the risk of hyperuricemia, using data from a nation-wide prospective cohort study conducted among middle-aged and elder Chinese population. Moreover, we evaluated the predictive power of different IR indicators for the development of hyperuricemia.

## 2. Materials and Methods

### 2.1. Study Design and Population

**Figure 1:**
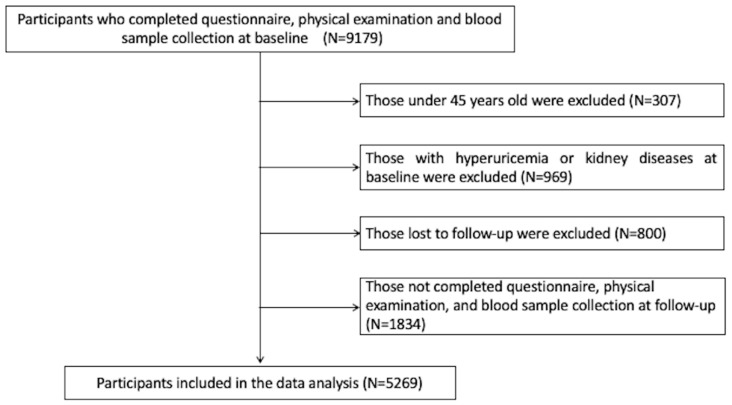
Flowchart for selecting the study population from the database of CHARLS.

We utilized the data from the China Health and Retirement Longitudinal Study (CHARLS), a national prospective cohort study conducted among middle-aged and older Chinese individuals (aged ≥ 45 years). A detailed description of CHARLS has previously been presented elsewhere [24]. In a nutshell, 17,708 participants and their spouses were recruited by a four-stage probabilistic sampling approach in 2011. All participants are followed up every two to three years, with four follow-ups having been completed to date. Physical condition data and laboratory blood tests results were obtained at wave 1 (2011) and wave 3 (2013). In the present study, data from waves 1 and 3 were utilized, on account of the accessibility of laboratory blood test data, therefore, 9179 participants were potentially qualified. After excluding individuals aged <45 years (*n* = 307), those with hyperuricemia or kidney diseases at baseline (*n* = 969), those lost to follow-up (*n* = 800), and those without complete information at follow-up (*n* = 1834), a total of 5269 subjects were eligible for the current study (Figure 1).

### 2.2. Data Collection and Measurement

The procedure of data collection for CHARLS included a face-to-face questionnaire interview, assays of venous blood and a physical examination. Using a questionnaire interview, demographic information, self-assessed health status and functioning, and health-related behaviors were collected. Anthropometry data were obtained by trained investigators using standard methods. Participants’ blood pressures were measured three times on their left arm, and the average of those three readings was recorded as the systolic and diastolic blood pressures (SBP and DBP). Fasting blood samples were collected from participants by professional medical staff. Fasting plasma glucose (FPG), total cholesterol (TC), low-density lipoprotein cholesterol (LDL-C), high-density lipoprotein cholesterol (HDL-C) and triglyceride (TG) levels were determined using an enzymatic colorimetric test. High-performance liquid chromatography was used to measure hemoglobin A1c (HbA1c) levels. Creatinine, blood urea nitrogen (BUN), and serum uric acid (SUA) were assayed using the rate-blanked and compensated Jaffe creatinine method, Enzymatic UV method with Urease, and UA Plus method, respectively.

### 2.3. Definitions of Covariates

In this study, the demographic factors included age, sex, residential location, marital status, education status, and alcohol drinking and smoking status. Hypertension was diagnosed when the SBP exceeded 140 mmHg and/or the DBP exceeded 90 mmHg [25]. Diabetes was defined when fasting plasma glucose ≥ 126 mg/dL (7.0 mmol/L) and/or a HbA1c ≥ 6.5% [26]. Dyslipidemia was determined according to the 2016 Chinese guidelines for the management of dyslipidemia [27], as a TC level of ≥240 mg/dL (6.2 mmol/L), a TG level of ≥200 mg/dL (2.3 mmol/L), a LDL-C level of ≥160 mg/dL (4.1 mmol/L), and/or a HDL-C level of ≤40 mg/dL (1.0 mmol/L). Additionally, self-reported hypertension, diabetes, and dyslipidemia were also employed for diagnosis. Cardiovascular disease was defined as participants’ self-reports of ever having been diagnosed with heart attack and/or stroke by doctors. Body mass index (BMI) was calculated as weight in kilograms divided by the square of height in meters (kg/m^2^). Estimated glomerular filtration rate (eGFR) was calculated using the CKD-EPI formula [28].

### 2.4. Definitions of TyG Index, TG/HDL-C Ratio, METS-IR, TyG-BMI and Hyperuricemia

The following formulas were used to calculate the non-insulin-based IR indices, including the TyG index, TG/HDL-C ratio, METS-IR and TyG-BMI [20,29,30]:

(1) TyG=ln[(TG(mg/dL)×FPG(mg/dL)/2)]

(2) TG/HDL−C=TG(mg/dL)/HDL−C(mg/dL)

(3) TyG−BMI=ln[(TG(mg/dL)×FPG(mg/dL)/2]×BMI

(4) $$METS-IR=ln[2\times FPG(mg/dL)+TG(mg/dL)]\times BMI(kg/m^{2})/ln[HDL-C(mg/dL)]$$

To analyze the changes in TyG index, TG/HDL-C ratio, TyG-BMI, METS-IR throughout follow-up, participants were classified into high and low levels based on the cut-off points derived from the analysis of restricted cubic spline curves. The cut-off values for TyG index, TG/HDL-C ratio, TyG-BMI, and METS-IR were defined as 8.60, 2.27, 200.68, and 34.41, respectively. The change in each IR surrogate during the follow-up period from 2011 to 2015 was categorized into four patterns: (1) Low–low: consistently low levels during follow-up; (2) Low-to-high: low levels at baseline but changed to high levels at follow-up; (3) High-to-low: high levels at baseline but changed to low levels at follow-up; (4) High–high: consistently high levels throughout follow-up. Hyperuricemia was characterized as a serum UA level of ≥7 mg/dL for males and ≥6 mg/dL for females [31].

### 2.5. Statistical Analysis

Continuous variables that were normally distributed were measured using mean and standard deviation (SD), otherwise the median and interquartile range (IQR) were used. Frequencies and percentage were calculated for categorical variables. Data normality was verified using Anderson–Darling normality test. Between-group comparisons were conducted using Students’ *t*-test, Chi-square test or Wilcoxon rank-sum test. We investigated the association between baseline surrogates of IR at CHARLS 2011 and incident hyperuricemia at CHARLS 2015 using multivariate logistic regression, with the results shown as odds ratio (OR) and corresponding 95% confidential interval (95% CI). The standardized beta coefficients in the multivariate logistic regression model were calculated using the approach described by Menard et al. [32]. Ordinal categorical variables were considered as continuous variables to examine linear trends in the logistic regression model. To examine the predictive power of different non-insulin-based IR indicators for hyperuricemia, the area under the curve (AUC) was calculated through the receiver operating characteristic (ROC) curve. The DeLong test was utilized to compare the areas under ROC curves [33]. Restricted cubic spline regression was performed to analyze the potential nonlinearity association between these indices and hyperuricemia. To further investigate the longitudinal influence of non-insulin-based IR indices variation on hyperuricemia incidence, four patterns of indices variations were classified: low–low level, low-to high level, high-to-low level, and high–high level. We calculated OR for low-to-high group, high-to-low group and high–high group with the low–low group as a reference using multivariate logistic regression. Stratified analyses were conducted by gender (male and female). A *p*-value of less than 0.05 indicated a statistically significant difference. The analysis of data was conducted utilizing R software, version 4.2.1 (R Foundation for Statistical Computing)

## 3. Results

### 3.1. Baseline Characteristics

Table 1 displays the baseline features of 5269 individuals categorized according to their incident hyperuricemia status. After four years of follow-up, 517 (9.81%) cases developed incident hyperuricemia. Individuals with hyperuricemia had a higher likelihood of being of advanced age, male, drinkers, and having conditions such as hypertension, diabetes, cardiovascular disease, and dyslipidemia. Additionally, these participants had higher baseline levels of TyG, TG/HDL-C, TyG-BMI, and METS-IR (Table 1).

### 3.2. Association between Baseline Insulin Resistance Surrogates and Hyperuricemia Risk

Through multivariate logistic regression analysis (Model 3), a significant linear trend towards an increased risk of hyperuricemia as TyG, TG/HDL-C, METS-IR, and TyG-BMI levels increased was demonstrated (all *p* for trend < 0.001). When compared to the lowest quartile of TyG, the adjusted odds ratios (ORs) for developing hyperuricemia in the second, third, and fourth quartiles were 1.49 (95% CI: 1.09–2.04), 1.57 (95% CI: 1.15–2.16), and 2.39 (95% CI: 1.72–3.34), respectively (Model 3). Similarly, those in the second, third, and fourth METS-IR quartiles were more likely to develop hyperuricemia than those in the first quartiles. When TG/HDL-C was utilized as the predictor variable, individuals in the third and fourth quartiles showed a significantly greater risk of hyperuricemia than those in the first quartile (OR = 1.57; 95% CI: 1.16–2.14, and OR = 2.42; 95% CI: 1.74–3.38, respectively) (Model 3). The findings also indicated that people at the highest quartile had a 3.35-fold (95% CI 2.40–4.73) higher risk of developing hyperuricemia compared to those in the lowest quartile of TyG-BMI (Table 2). The beta coefficients and corresponding standardized beta coefficient in model 3 are shown in Table 2 and Table 3. Among all variables significantly associated with hyperuricemia in the model 3, the standardized beta coefficients of the four IR indicators were the largest, expect for creatinine. The associations between the four IR surrogates and the risk of hyperuricemia, stratified by gender, are illustrated in Figure S1.

### 3.3. ROCs of TyG Index, TyG/HDL-C Ratio, METS-IR, TyG-BMI for Hyperuricemia

**Figure 2:**
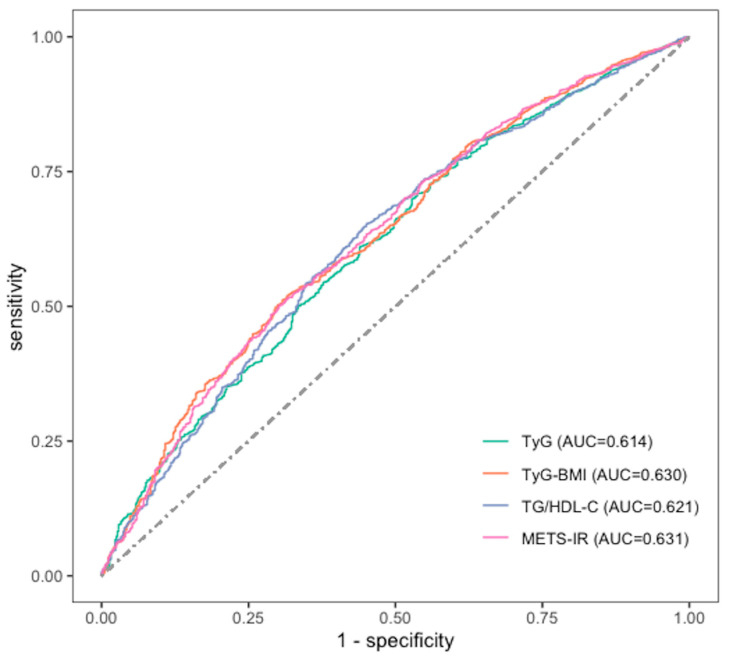
Receiver operating characteristic (ROC) curves for incident hyperuricemia comparing TyG, TG/HDL-C, TyG-BMI, and METS-IR.

Figure 2 illustrates the ROC curve and the AUC values for TyG index, TG/HDL-C ratio, METS-IR and TyG-BMI in the prediction of hyperuricemia during four-year follow-up. The highest AUC was found for METS-IR (AUC = 0.631), followed by TyG-BMI (AUC = 0.630), TG/HDL-C (AUC = 0.621), and TyG (AUC = 0.614). The AUC values of these four indicators were higher for females than males (Figure S2 and Table S1). The DeLong test indicated no statistically significant difference between the predictive abilities of these four indexes for hyperuricemia (all *p* > 0.05) in total participants and among males. In females, METS-IR showed higher prediction ability for incident hyperuricemia than TyG using Delong tests (*p* = 0.038) (Table S1). The threshold values, sensitivity, and specificity of these four indicators in predicting hyperuricemia are displayed in Table S1.

### 3.4. Restricted Cubic Spline Regression

**Figure 3:**
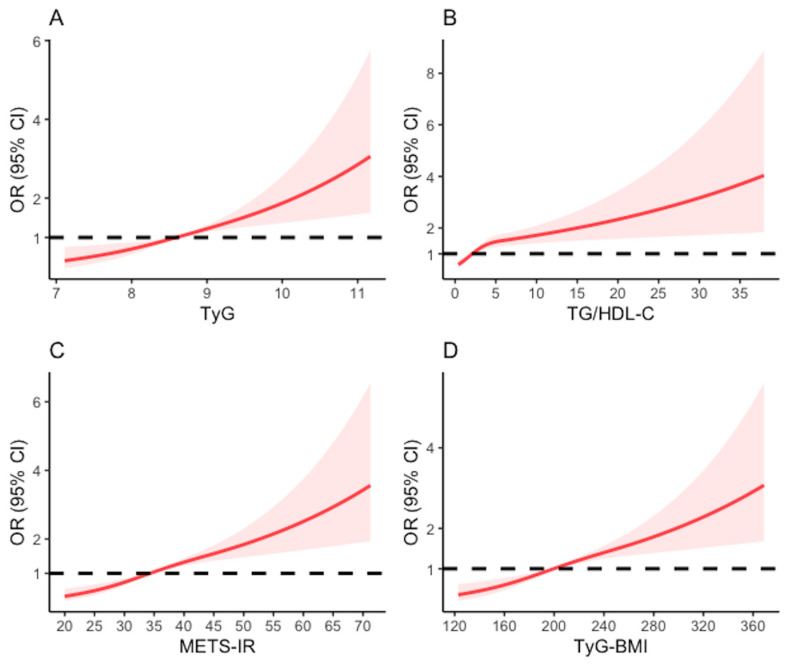
The associations of (**A**) TyG index, (**B**) TG/HDL-C, (**C**) METS-IR, and (**D**) TyG-BMI at baseline with hyperuricemia in Chinese middle-aged population. Data were fitted by logistic regression models of the restricted cubic spline with 3 knots adjusting for age, sex, resident, education level, married status, smoking history, drinking history, hypertension, diabetes cardiovascular disease, dyslipidemia, total cholesterol, blood urea nitrogen, creatinine, glycated hemoglobin, and C-reactive protein.

Figure 3 illustrates the utilization of restricted cubic splines to visualize and analyze the dose-relationship between four IR surrogates and hyperuricemia in the middle-aged and elderly Chinese population. After adjusting for multiple variables, we observed nonlinear relationships for TG/HDL-C (*p* for nonlinearity <0.001) and METS-IR (*p* for nonlinearity = 0.031). The risk of hyperuricemia was relatively stable at TG/HDL-C or METS-IR levels below 2.27 or 34.41, respectively, after which it began to increase rapidly. With increasing TyG and TyG-BMI, we observed significant linear relationships with the risk of hyperuricemia (*p* for nonlinearity = 0.733 and 0.086, respectively), with the cut-off values of 8.60 and 200.68, respectively.

### 3.5. Association between the Variation of TyG, TG/HDL-C, METS-IR, TyG-BMI and Hyperuricemia

Table 4 presents the correlation of hyperuricemia risk with different IR indicator variation patterns. Compared to those who maintained low TyG, individuals with variation patterns of low-to-high TyG or high-to-high TyG both increased the risk of developing hyperuricemia (OR = 1.71, 95%CI 1.24–2.36; OR = 2.28, 95%CI: 1.73–3.02, respectively) (Model 3). Likewise, variation patterns of low-to-high TG/HDL-C and high–high TG/HDL-C were associated with increased risks of hyperuricemia, with adjusted ORs of 1.53 (95% CI: 1.12–2.08) and 2.34 (95% CI: 1.80–3.06), respectively. In comparison with individuals who maintained low METS-IR levels during follow-up, the risk of hyperuricemia was higher in those with variation patterns of high-to-high (OR = 2.19, 95% CI: 1.70–2.83), high-to-low (OR = 1.96, 95% CI: 1.32–2.87), and low-to-high (OR = 1.62, 95% CI: 1.12–2.30). Results also showed that the maintaining a high level of TyG-BMI was associated with a 2.20-fold greater risk of hyperuricemia than maintaining a low level, and a 1.63-fold and a 1.58-fold higher risk were shown in those under changing patterns of high-to-low and low-to-high, respectively. Furthermore, stratified analyses indicated that maintaining high levels of four insulin surrogates significantly increased the risk of hyperuricemia in both genders compared to maintaining low pattern (all *p* < 0.05), with higher adjusted ORs for females than males (Figure S3).

## 4. Discussion

In this national cohort study, positive associations were identified between four non-insulin-based IR indicators and risk of hyperuricemia among middle-aged and older Chinese individuals. These four indexes (TyG, TG/HDL-C, TyG-BMI, METS-IR) demonstrated comparable predictive ability for hyperuricemia. Additionally, variations from low to high levels and persistent high levels of IR surrogates could also increase the risk of hyperuricemia. Furthermore, IR surrogates were also significantly associated with hyperuricemia risk in both genders, with a stronger association observed in females compared to males.

Our results are in accordance with prior research indicating the deleterious effects of elevated indictors for IR on the development of hyperuricemia. Wang et al. [22] carried out a cross-sectional study utilizing data from National Health and Nutrition Examination Survey (NHANES) in the United States and reported a positive correlation of TyG, TyG-BMI, TG/HDL-C and METS-IR with hyperuricemia. Liu et al. and colleagues reported similar findings in another study [23], which compared three surrogates of IR, including TyG, TG/HDL-C and METS-IR, using the data from a cross-sectional survey conducted in eastern China. The cross-sectional design hindered the ability to establish a causal relationship among insulin resistance and hyperuricemia. To explore the potential cause-and-effect relationship, longitudinal data is needed. The findings from a 6-year retrospective cohort [34] and a 4-year prospective study [20] corroborated our results. Overall, our study provides evidence about the causative effect of insulin resistance on hyperuricemia development. However, the predictive power of these IR surrogates for hyperuricemia varied among different studies. In our study, there were no significant differences in the predictive power of these four indicators of IR in predicting hyperuricemia, while another study conducted among U.S. non-diabetic adults showed that TyG-BMI and METS-IR had better recognition of hyperuricemia than TyG and TG/HDL-C [22]. This could be attributed to differences in research design, inclusion of covariates, and ethnic population [30]. Our results provide evidence that the four surrogates for IR have similar predictive performance in screening people at high risk for hyperuricemia in the Chinese middle-aged and elderly population.

Previous studies have emphasized the importance of IR surrogates. In our study, we identified four patterns of variations from the years 2011 to 2015, and people with maintained high and low-to-high levels of IR surrogates had an increased risk of incident hyperuricemia compared to those who maintained low patterns. The results suggest that participants with elevated or maintained high levels of insulin resistance were more likely to develop hyperuricemia. Further intervention studies could explore ways of reducing and maintaining low levels of IR to prevent incident hyperuricemia. Furthermore, people with high-to-low variation patterns showed a higher risk of hyperuricemia in comparison to those with maintained low variation patterns. These results indicate that a high IR state increases the risk of developing hyperuricemia. Early detection and intervention are crucial in preventing irreversible hyperuricemia risks that may arise from high levels of IR. This also emphasizes the significance of monitoring insulin resistance levels in individuals at risk of developing hyperuricemia. Since our study only conducted a 4-year follow-up, longer follow-up times may be needed to observe the cumulative effect of insulin resistance on hyperuricemia.

Furthermore, we discovered that IR surrogates have a greater predictive ability on hyperuricemia in females compared to males. A study conducted among adults in the United States indicated similar findings, which revealed that the association between IR surrogates and hyperuricemia was more pronounced in female patients [22]. Interestingly, the similar findings have been also noted in the associations between IR indicators and the risk of type 2 diabetes mellitus, cardiovascular disease, and non-alcoholic fatty liver disease [35,36,37]. The effects of IR on incident hyperuricemia may vary across gender, possibly because females have distinct sex hormones and adipokines that make them more responsive to insulin than male [38]. Studies have demonstrated the protective impact of estrogen on IR and glucose production through estrogen receptor-α [37]. The decrease in estrogen levels following menopause could cause an imbalance in glucose metabolism, increasing the risk of women developing hyperuricemia. Since healthy women have higher fat mass than men, they have higher levels of circulating free fatty acids and intramuscular fat content, which may increase the susceptibility to IR in women [39]. Thus, the IR surrogates may have a more prominent value in predicting the occurrence of hyperuricemia in women. In the future, it may be worthwhile to focus on applying these indices in women to predict hyperuricemia occurrence.

There is an accumulating body of research indicating the associations between IR and hyperuricemia. However, the findings are still under dispute, especially regarding the chicken-and-egg dilemma between UA and IR. McCormick et al. used the bidirectional Mendelian randomization to find that hyperinsulinemia may induce hyperuricemia, but not vice versa [15]. Zhu et al. also used a Mendelian randomization approach, but indicated that increased UA levels preceded IR [40]. Interestingly, Hu et al. revealed a correlation between increased UA levels and a higher risk of IR through Mendelian randomization analysis, but no significant causal association was found [41]. The inconsistencies of the above results might be due to the different type of population enrolled, hereditary factor, as well as the techniques that used for testing IR [38]. Several biological mechanisms have been proposed to explain the potential association between IR and hyperuricemia. Studies have shown that the development of hyperuricemia may be associated with increased expression of urate transporter 1 (URAT1) and glucose transporter 9 (GLUT9), as well as glycolytic disturbances caused by IR [42]. In particular, IR could affect renal UA transport, resulting in increased UA reabsorption [13]. Furthermore, IR may result in an increased rate of fat breakdown and reduced activity of lipoprotein lipase in adipose tissue [43], which in turn could lead to hyperlipemia and excessive the production of UA.

The present study is a nationally representative investigation that evaluated the longitudinal effect of three IR surrogates on the development of hyperuricemia among the general Chinese population. Our study utilized the data from CHARLS, which has a wide geographic coverage, strict implementation procedures, and nationwide samples, thereby ensuring our conclusions are nationally representative. This is a novel study investigating the correlations between changes in IR surrogates and hyperuricemia. Another prominent feature of this study is that it compares the predictive ability of TyG, TG/HDL-C, TyG-BMI, and METS-IR based on a longitudinal dataset. Despite the above strengths of this study, there are also several limitations that should be considered. Until now, CHALRS has only provided for wave 1 (2011) and wave 3 (2015); hence, the follow-up duration in the current study was four years. Further verification of the results could be carried out by implementing a longer follow-up. Additionally, due to the original design of CHARLS, even though our analysis has controlled and adjusted for a variety of covariates, there might still be some additional uncollected confounders, such as dietary factors like intake of meat, vegetables and dairy.

## 5. Conclusions

In conclusion, the levels of IR surrogates, including TyG, TG/HDL-C, TyG-BMI and METS-IR, are associated with the risk of hyperuricemia among the middle-aged and older Chinese population. There were significant correlations between variations of these predictors’ status and the risk of hyperuricemia, with stronger associations being found in women. Monitoring insulin resistance surrogates could serve as a useful method to anticipate the risk of hyperuricemia, and may help to prevent this condition from developing in middle-aged and older Chinese individuals. Further research into the underlying mechanisms linking insulin resistance and hyperuricemia, and for developing effective strategies for preventing and treating these conditions are needed.

## Figures and Tables

**Table 1:** Baseline characteristics of participants stratified by incident hyperuricemia status.

Variables	Overall	Hyperuricemia		*p* Value
		No	Yes	
Overall, *n* (%)	5269 (100.00)	4752 (90.19)	517 (9.81)	-
Age (years, mean ± SD)	58.58 ± 8.61	58.41 ± 8.53	60.11 ± 9.15	<0.001
Gender, *n* (%)				
Male	2386 (45.28)	2119 (44.59)	267 (51.64)	0.003
Female	2883 (54.72)	2633 (55.41)	250 (48.36)	
Residence, *n* (%)				
Rural	3540 (67.19)	3210 (67.55)	330 (63.83)	0.097
Urban	1729 (32.81)	1542 (32.45)	187 (36.17)	
Education status, *n* (%)				
Illiterate	1509 (28.64)	1374 (28.91)	135 (26.11)	0.369
Primary school and below	2234 (42.40)	2003 (42.15)	231 (44.68)	
Middle school and above	1526 (28.96)	1375 (28.94)	151 (29.21)	
Marital status, *n* (%)				
Single	4704 (89.28)	4250 (89.44)	454 (87.81)	0.291
Married/cohabiting	565 (10.72)	502 (10.56)	63 (12.19)	
Smoking history, *n* (%)				
Non-smoker	3272 (62.10)	2967 (62.44)	305 (58.99)	0.138
Smoker	1997 (37.90)	1785 (37.56)	212 (41.01)	
Drinking status, *n* (%)				
Non-drinker	3277 (62.19)	2993 (62.98)	284 (54.93)	<0.001
Drinker	1992 (37.81)	1759 (37.02)	233 (45.07)	
Hypertension, *n* (%)				
No	3212 (60.96)	2988 (62.88)	224 (43.33)	<0.001
Yes	2057 (39.04)	1764 (37.12)	293 (56.67)	
Diabetes mellitus, *n* (%)				
No	4428 (84.04)	4010 (84.39)	418 (80.85)	0.037
Yes	841 (15.96)	742 (15.61)	99 (19.15)	
Cardiovascular disease, *n* (%)				
No	4587 (87.06)	4162 (87.58)	425 (82.21)	<0.001
Yes	682 (12.94)	590 (12.42)	92 (17.79)	
Dyslipidemia, *n* (%)				
No	3024 (57.39)	2801 (58.94)	223 (43.13)	<0.001
Yes	2245 (42.61)	1951 (41.06)	294 (56.87)	
BMI, kg/m^2^, mean ± SD	23.55 ± 3.70	23.41 ± 3.67	24.75 ± 3.75	<0.001
FPG, mg/dL, median (IQR)	102.42 (94.5, 112.68)	102.24 (94.32, 112.32)	104.4 (95.76, 116.28)	0.001
HDL-C, mg/dL, median (IQR)	49.48 (40.59, 59.92)	49.87 (40.98, 60.31)	45.62 (37.11, 54.51)	<0.001
TG, mg/dL, median (IQR)	105.32 (74.34, 152.22)	102.66 (73.46, 147.79)	130.98 (87.61, 194.7)	<0.001
Total cholesterol, mg/dL, median (IQR)	190.59 (167.01, 214.95)	190.01 (166.62, 214.56)	195.62 (173.97, 219.59)	<0.001
HbA1c, %, median (IQR)	5.1 (4.9, 5.4)	5.1 (4.9, 5.4)	5.2 (4.9, 5.5)	0.002
Creatinine, mg/dL, median (IQR)	0.75 (0.64, 0.86)	0.73 (0.63, 0.85)	0.80 (0.71, 0.94)	<0.001
C-reactive protein, mg/L, median (IQR)	0.98 (0.54, 2.02)	0.95 (0.52, 1.94)	1.31 (0.71, 2.70)	<0.001
eGFR, ml/min per 1.73 m^2^, median (IQR)	95.87 (86.2, 102.78)	96.39 (87.16, 103.19)	90.41 (79.30, 98.62)	<0.001
BUN, mg/dL, median (IQR)	14.99 (12.49, 18.04)	14.93 (12.44, 18.04)	15.55 (13.08, 17.87)	0.017
SUA, mg/dL, median (IQR)	4.17 (3.51, 4.94)	4.08 (3.45, 4.79)	5.22 (4.57, 5.93)	<0.001
TyG, median (IQR)	8.59 (8.22, 9.03)	8.57 (8.21, 9.00)	8.82 (8.42, 9.30)	<0.001
TG/HDL-C, median (IQR)	2.11 (1.32, 3.53)	2.05 (1.29, 3.39)	2.94 (1.71, 4.68)	<0.001
METS-IR, median (IQR)	34.31 (29.84, 40.17)	33.91 (29.59, 39.60)	37.67 (32.73, 44.38)	<0.001
TyG-BMI, median (IQR)	199.52 (176.27, 229.01)	197.71 (175.18, 225.77)	213.68 (191.58, 250.4)	<0.001

IQR, interquartile range; SD, standard deviation; FPG, fasting plasma glucose; TG, triglyceride; HDL-C, high-density lipoprotein cholesterol; LDL-C, low-density lipoprotein cholesterol; HbA1c, glycated hemoglobin, BUN, blood urea nitrogen; eGFR, estimated glomerular filtration rate; SUA, serum uric acid; TyG, triglyceride–glucose index; METS-IR, metabolic score for insulin resistance; TG/HDL-C, triglyceride-to-high-density-lipoprotein-cholesterol ratio; TyG-BMI, TyG with body mass index.

**Table 2:** Odds ratio of incident hyperuricemia associated with TyG, TG/HDL-C, METS-IR and TyG-BMI in middle-aged and older Chinese population.

Variables	No. of Cases (%)	OR (95% CI)				β	β_s_
		Crude Model	Model 1	Model 2	Model 3		
TyG							
Q1	73 (5.54)	1.00	1.00	1.00	1.00	-	-
Q2	113 (8.49)	1.58 (1.17, 2.15)	1.62 (1.20, 2.22)	1.51 (1.11, 2.07)	1.49 (1.09, 2.04)	0.396	0.035
Q3	132 (9.73)	1.84 (1.37, 2.49)	1.92 (1.43, 2.61)	1.67 (1.23, 2.28)	1.57 (1.15, 2.16)	0.454	0.040
Q4	206 (15.49)	3.13 (2.38, 4.16)	3.33 (2.52, 4.44)	2.64 (1.92, 3.67)	2.39 (1.72, 3.34)	0.870	0.081
*p* for trend		<0.001	<0.001	<0.001	<0.001	-	-
TyG change, per SD increase	528 (9.79)	1.45 (1.33, 1.57)	1.47 (1.36, 1.61)	1.40 (1.25, 1.57)	1.36 (1.22, 1.54)	0.312	0.010
TG/HDL-C							
Q1	78 (5.91)	1.00	1.00	1.00	1.00	-	-
Q2	99 (7.45)	1.28 (0.94, 1.75)	1.32 (0.97, 1.80)	1.27 (0.93, 1.73)	1.24 (0.91, 1.71)	0.218	0.019
Q3	135 (10.03)	1.77 (1.33, 2.38)	1.87 (1.4, 2.51)	1.65 (1.22, 2.24)	1.57 (1.16, 2.14)	0.451	0.039
Q4	213 (15.88)	3.00 (2.30, 3.96)	3.25 (2.48, 4.31)	2.58 (1.86, 3.59)	2.42 (1.74, 3.38)	0.884	0.082
*p* for trend		<0.001	<0.001	<0.001	<0.001	-	-
TG/HDL-C change, per SD increase	528 (9.79)	1.27 (1.19, 1.36)	1.28 (1.20, 1.38)	1.19 (1.10, 1.28)	1.20 (1.11, 1.30)	0.184	0.004
METS-IR							
Q1	69 (5.24)	1.00	1.00	1.00	1.00	-	-
Q2	102 (7.52)	1.47 (1.07, 2.03)	1.59 (1.15, 2.19)	1.49 (1.08, 2.06)	1.49 (1.08, 2.07)	0.401	0.037
Q3	143 (10.86)	2.20 (1.64, 2.99)	2.52 (1.87, 3.44)	2.12 (1.55, 2.92)	2.09 (1.52, 2.88)	0.735	0.066
Q4	210 (15.64)	3.36 (2.54, 4.49)	4.06 (3.04, 5.49)	3.03 (2.18, 4.25)	2.89 (2.07, 4.07)	1.061	0.101
*p* for trend		<0.001	<0.001	<0.001	<0.001	-	-
METS-IR change, per SD increase	528 (9.79)	1.49 (1.38, 1.62)	1.58 (1.45, 1.72)	1.44 (1.30, 1.59)	1.43 (1.29, 1.58)	0.356	0.010
TyG-BMI							
Q1	63 (4.78)	1.00	1.00	1.00	1.00	-	-
Q2	101 (7.67)	1.47 (1.07, 2.03)	1.82 (1.32, 2.53)	1.71 (1.23, 2.39)	1.67 (1.20, 2.34)	0.512	0.048
Q3	144 (10.93)	2.20 (1.64, 2.99)	2.88 (2.12, 3.97)	2.40 (1.74, 3.34)	2.25 (1.63, 3.14)	0.812	0.075
Q4	209 (15.87)	3.36 (2.54, 4.49)	4.78 (3.54, 6.53)	3.65 (2.62, 5.13)	3.35 (2.40, 4.73)	1.209	0.116
*p* for trend		<0.001	<0.001	<0.001	<0.001	-	-
TyG-BMI change, per SD increase	528 (9.79)	1.51 (1.39, 1.64)	1.62 (1.48, 1.77)	1.47 (1.33, 1.63)	1.43 (1.29, 1.59)	0.361	0.011

Model 1: adjusted for age, sex, resident, education level, married status, smoking history, drinking history. Model 2: adjusted for all the factors in model 1 and hypertension, diabetes, cardiovascular disease, dyslipidemia. Model 3: adjusted for all the factors in model 2 and total cholesterol, blood urea nitrogen, creatinine, glycated hemoglobin, and C-reactive protein. β, Beta coefficient; β_s_, Standardized beta coefficient. β and β_s_ are the estimated values of parameters in Model 3.

**Table 3:** Estimated values of covariate parameters in the logistic regression model for the association between insulin resistance surrogates and hyperuricemia.

	TyG Model			TG/HDL-C Model			METS-IR Model			TyG-BMI Model		
	β	β_s_	*p*	β	β_s_	*p*	β	β_s_	*p*	β	β_s_	*p*
Age	0.012	0.000	0.067	0.012	0.000	0.047	0.016	0.000	0.011	0.018	0.000	0.005
Sex	0.101	0.009	0.526	0.134	0.012	0.402	0.139	0.012	0.387	0.098	0.009	0.540
Resident	−0.015	−0.001	0.885	−0.029	−0.002	0.775	−0.073	−0.004	0.479	−0.072	−0.004	0.486
Education status	0.033	0.001	0.660	0.035	0.001	0.638	0.016	0.001	0.834	0.015	0.001	0.838
Married status	0.017	0.001	0.915	0.026	0.002	0.866	0.068	0.006	0.660	0.070	0.006	0.652
Smoking history	−0.189	−0.014	0.152	−0.194	−0.014	0.142	−0.117	−0.009	0.376	−0.091	−0.007	0.495
Drinking history	0.304	0.020	0.009	0.330	0.021	0.005	0.332	0.021	0.004	0.306	0.020	0.009
Hypertension	0.593	0.032	0.000	0.590	0.032	0.000	0.509	0.028	0.000	0.488	0.027	0.000
Diabetes	−0.320	−0.026	0.030	−0.209	−0.017	0.146	−0.252	−0.020	0.082	−0.276	−0.022	0.056
Cardiovascular disease	0.133	0.010	0.303	0.133	0.009	0.305	0.102	0.007	0.430	0.111	0.008	0.394
Dyslipidemia	0.304	0.019	0.006	0.160	0.011	0.189	0.213	0.013	0.056	0.263	0.015	0.014
Total cholesterol	0.000	0.000	0.962	0.001	0.000	0.335	0.002	0.000	0.200	0.000	0.000	0.781
Blood urea nitrogen	−0.010	0.000	0.417	−0.008	0.000	0.494	−0.011	0.000	0.360	−0.011	0.000	0.359
Creatinine	2.284	0.399	0.000	2.286	0.399	0.000	2.327	0.409	0.000	2.297	0.405	0.000
Glycated hemoglobin	0.075	0.003	0.226	0.090	0.003	0.150	0.066	0.002	0.296	0.060	0.002	0.342
C-reactive protein	0.006	0.000	0.250	0.007	0.000	0.245	0.005	0.000	0.381	0.006	0.000	0.320

TyG model: logistic regression model for the association between the quartiles of TyG and hyperuricemia. TG/HDL-C model: logistic regression model for the association between the quartiles of TG/HDL-C and hyperuricemia. METS-IR model: logistic regression model for the association between the quartiles of METS-IR and hyperuricemia. TyG-BMI model: logistic regression model for the association between the quartiles of TyG-BMI and hyperuricemia. β, beta coefficient; βs, standardized beta coefficient; *p*, *p*-value in logistic regression model.

**Table 4:** Risk of incident hyperuricemia by different insulin resistance surrogates’ variations in middle-aged and older Chinese.

Variation Types During Follow-Up	No. of Cases (%)	OR (95% CI)			
		Crude Model	Model 1	Model 2	Model 3
TyG					
Low–Low	93 (5.59)	1.00	1.00	1.00	1.00
Low–High	79 (9.34)	1.74 (1.27, 2.38)	1.86 (1.36, 2.55)	1.71 (1.24, 2.35)	1.71 (1.24, 2.36)
High–Low	52 (7.18)	1.31 (0.91, 1.85)	1.34 (0.94, 1.90)	1.19 (0.82, 1.69)	1.12 (0.78, 1.61)
High–High	293 (14.40)	2.84 (2.24, 3.64)	3.11 (2.44, 4.01)	2.46 (1.88, 3.24)	2.28 (1.73, 3.02)
TG/HDL-C					
Low–Low	114 (5.79)	1.00	1.00	1.00	1.00
Low–High	76 (8.67)	1.54 (1.14, 2.08)	1.65 (1.21, 2.23)	1.54 (1.13, 2.08)	1.53 (1.12, 2.08)
High–Low	55 (9.11)	1.63 (1.16, 2.27)	1.72 (1.22, 2.40)	1.50 (1.05, 2.12)	1.46 (1.02, 2.07)
High–High	272 (14.96)	2.86 (2.28, 3.61)	3.13 (2.49, 3.97)	2.49 (1.91, 3.24)	2.34 (1.80, 3.06)
METS-IR					
Low–Low	123 (5.78)	1.00	1.00	1.00	1.00
Low–High	46 (8.70)	1.55 (1.08, 2.19)	1.71 (1.19, 2.42)	1.60 (1.11, 2.27)	1.62 (1.12, 2.30)
High–Low	43 (11.44)	2.11 (1.45, 3.01)	2.33 (1.59, 3.35)	1.95 (1.32, 2.83)	1.96 (1.32, 2.87)
High–High	305 (13.65)	2.58 (2.08, 3.22)	2.97 (2.37, 3.74)	2.26 (1.76, 2.91)	2.19 (1.70, 2.83)
TyG-BMI					
Low–Low	124 (5.90)	1.00	1.00	1.00	1.00
Low–High	49 (8.25)	1.43 (1.01, 2.01)	1.68 (1.18, 2.37)	1.58 (1.10, 2.22)	1.58 (1.11, 2.24)
High–Low	35 (10.39)	1.85 (1.23, 2.71)	2.06 (1.37, 3.04)	1.70 (1.12, 2.53)	1.63 (1.07, 2.44)
High–High	309 (13.80)	2.55 (2.06, 3.18)	3.08 (2.46, 3.89)	2.35 (1.83, 3.02)	2.20 (1.71, 2.83)

Variation types during follow-up, the definitions of Low–Low, Low–High, High–Low and High–High were listed as follow: Group Low–Low, maintaining low status during follow-up; Group Low–High, Low status at baseline turned to High status at follow-up; Group High–High, High status at baseline turned to Low status at follow-up; Group High–High, maintaining High status during follow-up. Model 1: adjusted for age, sex, resident, education level, married status, smoking history, drinking history. Model 2: adjusted for all the factors in model 1 and hypertension, diabetes, cardiovascular disease, dyslipidemia. Model 3: adjusted for all the factors in model 2 and total cholesterol, blood urea nitrogen, creatinine, glycated hemoglobin, and C-reactive protein.

## Data Availability

The current study’s dataset is available for public access on the following website: http://charls.pku.edu.cn/en, accessed on 16 January 2023.

## Supplementary Materials

The following supporting information can be downloaded at: https://www.mdpi.com/article/10.3390/nu15143139/s1, Figure S1: Association of (A) TyG, (B) TG/HDL-C, (C) METS-IR, and (D) TyG-BMI with risk of hyperuricemia by sex. Figure S2: Receiver operating characteristic (ROC) curves for incident hyperuricemia comparing TyG, METS-IR, TG/HDL-C, TyG-BMI in A (males) and (B) females. Figure S3: Risk of incident hyperuricemia by different insulin resistance surrogates’ variations in middle-aged and older Chinese. Table S1: Area under curve the receiver operating characteristic curve for identifying hyperuricemia with four insulin resistance surrogates.
