# Supplementary material for: The Association of Surrogates of Insulin Resistance with Hyperuricemia among Middle-Aged and Older Individuals: A Population-Based Nationwide Cohort Study
Source: Nutrients. 2023 Jul 14;15(14):3139. doi: 10.3390/nu15143139 (PMC10385684; doi:10.3390/nu15143139)
Supplement: Supplementary file 1 [file nutrients-15-03139-s001.zip › nutrients-2487857-supplementary.pdf]

Supplementary Files

Title: The association of surrogates of insulin resistance with hyperuricemia among middle-aged and older individuals: a population-based nationwide cohort study

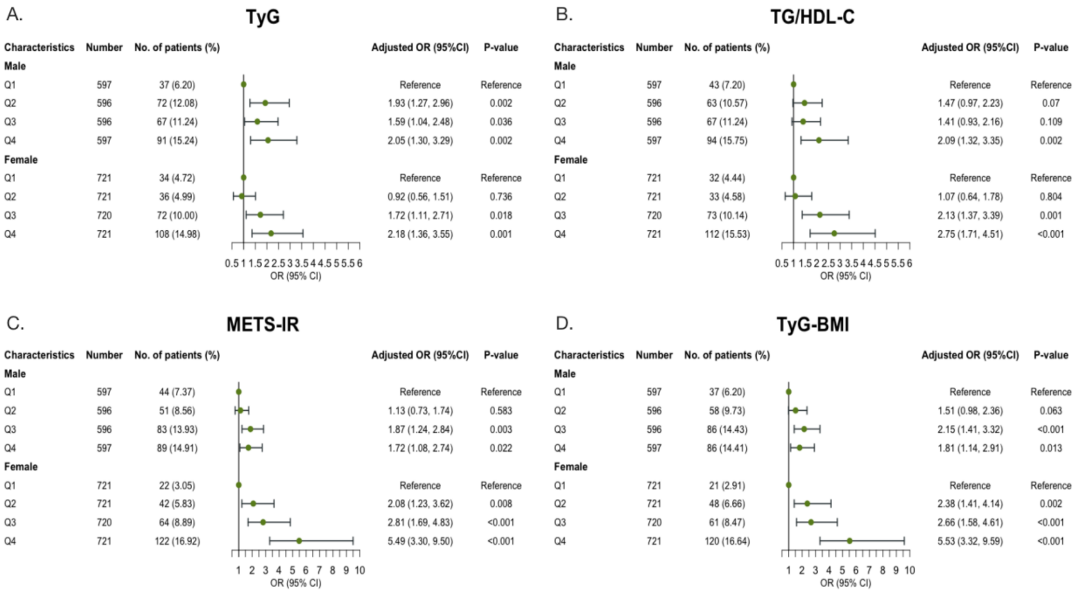

Figure S1. Association of (A) TyG, (B) TG/HDL-C, (C) METS-IR, and (D) TyG-BMI with risk of hyperuricemia by sex. Data were presented as n (%) and odds ratios (95% CI), and adjusted for age, resident, education level, married status, smoking history, drinking history, hypertension, diabetes, cardiovascular disease, dyslipidemia, total cholesterol, blood urea nitrogen, creatinine, glycated hemoglobin, and C-reactive protein.

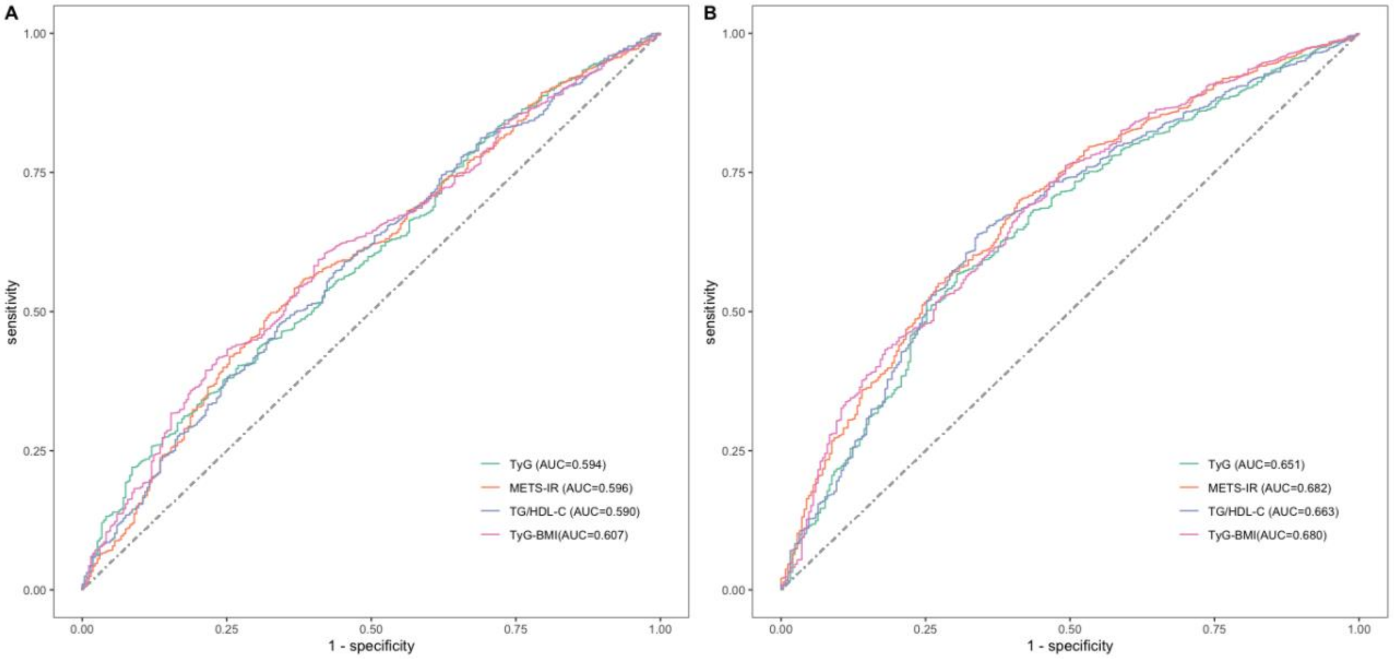

Figure S2. Receiver operating characteristic (ROC) curves for incident hyperuricemia comparing TyG, METS-IR, TG/HDL-C, TyG-BMI in A (males) and (B) females.

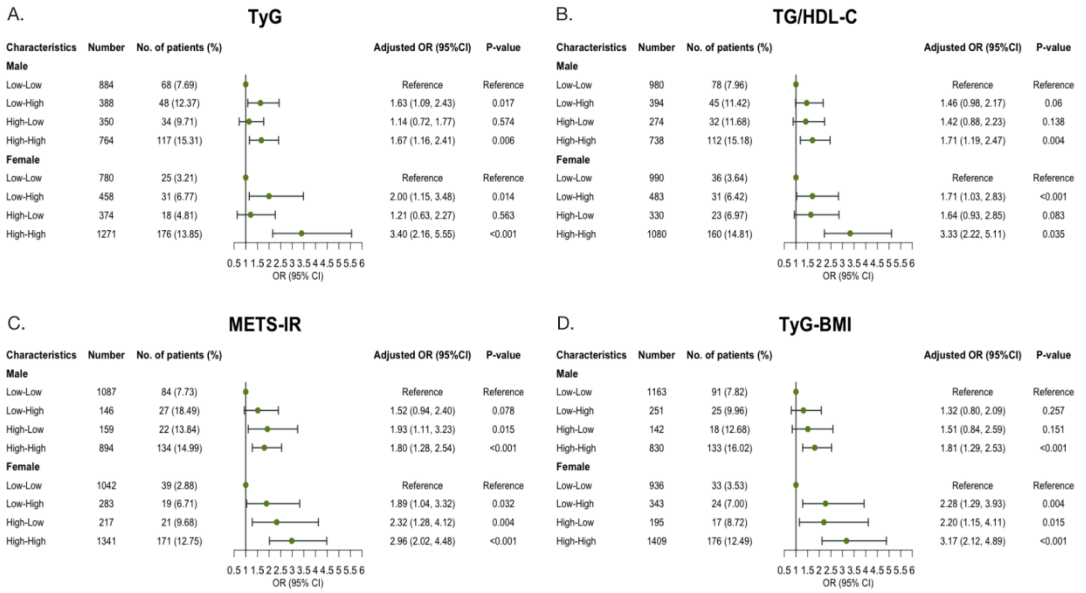

Figure S3. Risk of incident hyperuricemia by different insulin resistance surrogates' variations in middle-aged and older Chinese. Data were presented as n (%) and odds ratios (95% CI) stratified by sex. Covariates including age, resident, education level, married status, smoking history, drinking history, hypertension, diabetes, cardiovascular disease, dyslipidemia, total cholesterol, blood urea nitrogen, creatinine, glycated hemoglobin, and C-reactive protein were adjusted. (A) TyG, (B) TG/HDL-C, (C) METS-IR, (D) TyG-BMI.

Table S1. Area under curve the receiver operating characteristic curve for identifying hyperuricemia with four insulin resistance surrogates.

Variables	AUC (95% CI)	Optimal cutoff	Sensitivity	Specificity	*P* value*
**Total**					
TyG	0.614 (0.589, 0.640)	8.916	0.709	0.462	Reference
TG/HDL-C	0.621 (0.600, 0.647)	2.729	0.653	0.549	0.274
METS-IR	0.631 (0.606, 0.656)	33.923	0.501	0.698	0.122
TyG-BMI	0.630 (0.605, 0.655)	199.490	0.519	0.685	0.122
**Males**					
TyG	0.594 (0.559, 0.629)	8.137	0.258	0.880	Reference
TG/HDL-C	0.590 (0.554, 0.626)	1.845	0.478	0.659	0.632
METS-IR	0.596 (0.560, 0.632)	33.920	0.558	0.618	0.908
TyG-BMI	0.607 (0.572, 0.641)	199.829	0.605	0.581	0.378
**Females**					
TyG	0.651 (0.615, 0.686)	8.739	0.567	0.696	Reference
TG/HDL-C	0.663 (0.628, 0.698)	2.728	0.639	0.660	0.160
METS-IR	0.682 (0.648, 0.716)	39.123	0.700	0.588	0.038
TyG-BMI	0.680 (0.645, 0.714)	233.931	0.763	0.508	0.058

**P* was calculated by using the method described by De Long. et al. comparing the AUC of TyG index with the other insulin resistance related indexes.
